# Influence of Climate Change and Trophic Coupling across Four Trophic Levels in the Celtic Sea

**DOI:** 10.1371/journal.pone.0047408

**Published:** 2012-10-16

**Authors:** Valentina Lauria, Martin J. Attrill, John K. Pinnegar, Andrew Brown, Martin Edwards, Stephen C. Votier

**Affiliations:** 1 Marine Biology and Ecology Research Centre, Plymouth University, Plymouth, Devon, United Kingdom; 2 Marine Institute, Plymouth University, Plymouth, Devon, United Kingdom; 3 Centre for Environment Fisheries and Aquaculture Science (CEFAS), Lowestoft, Suffolk, United Kingdom; 4 Natural England, Peterborough, Cambridgeshire, United Kingdom; 5 Sir Alister Hardy Foundation for Ocean Science (SAHFOS), Plymouth, Devon, United Kingdom; University of Wales Swansea, United Kingdom

## Abstract

Climate change has had profound effects upon marine ecosystems, impacting across all trophic levels from plankton to apex predators. Determining the impacts of climate change on marine ecosystems requires understanding the direct effects on all trophic levels as well as indirect effects mediated by trophic coupling. The aim of this study was to investigate the effects of climate change on the pelagic food web in the Celtic Sea, a productive shelf region in the Northeast Atlantic. Using long-term data, we examined possible direct and indirect ‘bottom-up’ climate effects across four trophic levels: phytoplankton, zooplankton, mid-trophic level fish and seabirds. During the period 1986–2007, although there was no temporal trend in the North Atlantic Oscillation index (NAO), the decadal mean Sea Surface Temperature (SST) in the Celtic Sea increased by 0.66±0.02°C. Despite this, there was only a weak signal of climate change in the Celtic Sea food web. Changes in plankton community structure were found, however this was not related to SST or NAO. A negative relationship occurred between herring abundance (0- and 1-group) and spring SST (0-group: *p* = 0.02, slope = −0.305±0.125; 1-group: *p* = 0.04, slope = −0.410±0.193). Seabird demographics showed complex species–specific responses. There was evidence of direct effects of spring NAO (on black-legged kittiwake population growth rate: *p* = 0.03, slope = 0.0314±0.014) as well as indirect bottom-up effects of lagged spring SST (on razorbill breeding success: *p* = 0.01, slope = −0.144±0.05). Negative relationships between breeding success and population growth rate of razorbills and common guillemots may be explained by interactions between mid-trophic level fish. Our findings show that the impacts of climate change on the Celtic Sea ecosystem is not as marked as in nearby regions (e.g. the North Sea), emphasizing the need for more research at regional scales.

## Introduction

Human-induced climate change has profoundly impacted marine ecosystems across the globe. These impacts have had wide-ranging effects upon the physiology, distribution, phenology and abundance of species, resulting in long-term threats to biodiversity [1]. A key feature of these climate-induced impacts is a high degree of spatial heterogeneity [2],[3]. Understanding the nature of this variation is a key goal for assessing and mitigating the impacts of global climate change.

Environmental change may impact different trophic levels in varying ways [4], [5], such that marine food webs may be impacted both directly and indirectly [6]. Direct effects of climate change include the influence of temperature change, particularly for ectothermic organisms (i.e. fish and invertebrates), or extreme weather events, which can impact endothermic organisms [7]. Taken together these direct effects can influence physiology, morphology and behaviour, leading to a suite of emergent ecological responses [8]. Indirect effects are typically mediated via trophic coupling. This is normally manifested via bottom-up control where climate-mediated changes in the availability of lower trophic levels have knock on consequences for higher trophic levels [4], [9]. Marine higher trophic level predators can also govern the abundance of lower trophic levels by top-down control [10], or mid-trophic level species may exert both top-down and bottom-up effects in a process known as wasp-waist control [11]. It is still unclear, however, how the nature of these effects, as well as ecosystem responses, varies across regions [12], [13].

The aim of this study was to look for climate-related influences across four trophic levels in the Celtic Sea, a productive shelf region in the northeast Atlantic. This is an extremely important area in terms of fish and invertebrate biodiversity [14] and it supports a large community of apex predators in the form of seabirds [15] and marine mammals, as well as several important European fisheries [16]. Profound climate-mediated changes to the nearby North Sea have led to concerns about the long-term viability of certain populations of apex predators [4], [17] so a key question is to determine how climate change might be impacting the Celtic Sea food web. This region is quite different from the North Sea in terms of physical characteristics and oceanography (i.e. general circulation pattern, depth and sea temperature) [18], [19] and this may influence trophic responses to climate change.

We tested for direct and indirect effects of climate across four trophic levels. Direct climate effects at each level of the food web were expected via a significant correlation between abundance/biomass/demography and environmental variables: the North Atlantic Oscillation index (NAO) and Sea Surface Temperature (SST). In the North Atlantic variations in the NAO index have induced changes in the zooplankton abundance, distribution and community assemblage [2], [20], [21]. In addition, the NAO strongly influences the frequency of extreme weather events, which may directly impact some seabirds [7]. SST may have direct effects through changes to the biology and distribution of ectothermic fish and invertebrates [6].

Indirect effects were expected via bottom-up processes, characterised by a positive correlation between a measure of predator abundance/biomass/demography and prey. We also tested for potential top-down effects typified by a negative correlation between predator and prey. We modelled the Celtic Sea pelagic food web simplified into four trophic levels: four species of piscivorous seabird (black-legged kittiwake *Rissa tridactyla*, hereafter kittiwake; common guillemot *Uria aalge*, hereafter guillemot; razorbill *Alca torda* and Atlantic puffin *Fratercula artica*, hereafter puffin), pelagic fish (Atlantic herring *Clupea harengus*, hereafter herring), zooplankton and phytoplankton. For each trophic level, long-term data from 1986 to 2007 were collated and analysed along with measures of environmental conditions (SST and NAO).

## Materials and Methods

### Study area

**Figure 1:**
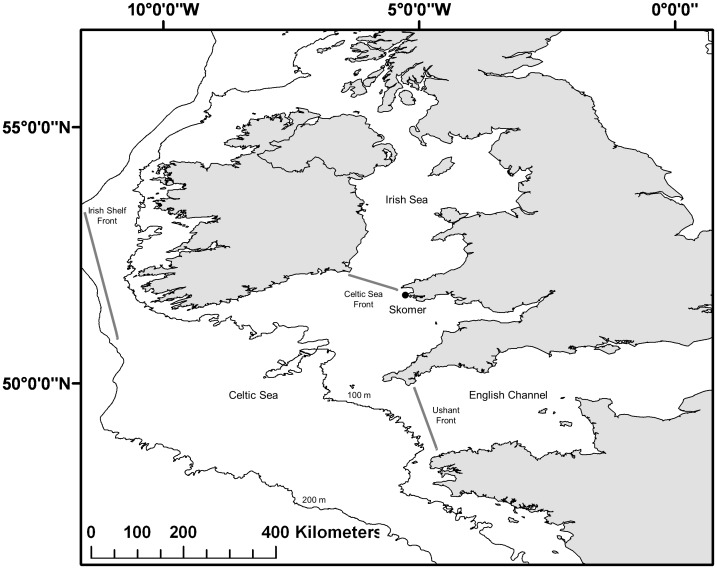
The Celtic Sea region (adapted from OSPAR, 2000). The Celtic Sea region considered in this study (49–53° N, 4–10°W). Including oceanographic features and the seabird colony investigated in this study (Skomer Island: 51°40N, 05°15W). The Irish Shelf Front occurs to the south and west of Ireland and exists all year-round. This front marks the boundary between waters of the shelf (often mixed vertically by the tide) and offshore North Atlantic waters. In addition there are two seasonal fronts systems which tend to develop during spring: the Celtic Sea front (dividing the Celtic Sea from the Irish Sea) and the Ushant Front, which develops from the coast of Brittany and extends to the western English Channel (dividing the Celtic Sea from the English Channel).

The Celtic Sea is an area of the northeast Atlantic continental shelf, southwest of the United Kingdom (Figure 1). It represents a transition zone between the Atlantic Ocean and coastal waters of the Bristol Channel and Irish Sea. There is a persistent north-westwards current running from Brittany to the Bristol Channel, as well as oceanographic fronts (the Irish Shelf, the Celtic Sea and Ushant fronts), which tend to inhibit lateral dispersal of phytoplankton [19].

### Phytoplankton

The annual phytoplankton bloom in the Celtic Sea typically occurs from April to October [22]. Continuous Plankton Recorder (CPR) data suggest a steady increase in phytoplankton over at least the last 20 years in the region [22]. Phytoplankton productivity and taxonomic composition in the Celtic Sea depend on water column structure: diatoms dominate areas with high nutrient content and display high rates of productivity, while dinoflagellates and microflagellates are found in stratified waters exhibiting lower rates of productivity. Productivity is reasonably high on the shelf with a rapid decrease west of the shelf break [22].

### Zooplankton

In the Celtic Sea the large copepod *Calanus helgolandicus* is an important component of the zooplankton community [23]; CPR data suggest an overall decline in the abundance of zooplankton with *Calanus* abundance falling below the long term mean in the region [22]. Long-term studies reveal that spatial patterns of zooplankton have changed significantly over the past 40 years, possibly as a result of climate-related reorganization of the zooplankton [24]. The ecological mechanism responsible for these changes remains unclear, however, and further analysis of CPR data in relation to environmental change is needed to clarify the situation.

### Pelagic fish

This region is particularly important for pelagic fish such as herring, sardine *Sardina pilchardus* and sprat *Sprattus sprattus*, and it is an important spawning ground for key migratory species notably mackerel *Scomber scombrus*, horse mackerel *Trachurus trachurus*, and blue whiting *Micromesistius potassou* [14], [25]. Like many coastal seas, the size-structure of the fish community has changed significantly over recent decades: there has been a decrease in the relative abundance of larger fish with a concomitant increase in the numbers of smaller fish [16]. Henderson (2007) [26] reports two main events in the 1980s and 1990s representing changes in the fish community composition, coinciding with climate-induced changes of plankton community in some regions of the North Atlantic [2].

### Seabirds

The Celtic Sea is an extremely important area for seabirds, supporting ∼300,000 breeding pairs of 15 species [15], including internationally important populations of northern gannet (*Morus bassanus*) and Manx shearwater (*Puffinus puffinus*), as well as nationally or regionally important populations of guillemot, lesser black-backed gull (*Larus fuscus*), herring gull (*Larus argentatus*), kittiwake, puffin and razorbill.

### Environmental variables

Two environmental predictors were used to test for direct and indirect effects of climate change: the North Atlantic Oscillation (NAO) and Sea Surface Temperature (SST). The description of the ecological mechanism associated with each climate predictor is summarised in Table 1.

**Table 1:** Potential climate effects for four trophic levels in the Celtic Sea.

Trophic level	Climate variable	Type of effect	Parameter related to climate variability	Suggested mechanism	Climate predictor used in this study	Reference
Phytoplankton	NAO	Direct	Abundance	Not defined possible effect of mixing waters	Winter NAO	[63], [64]
	SST	Direct	Abundance/distribution	Effect of nutrients availability, metabolic rates and water stratification	Winter SST	[65]
Zooplankton	NAO	Direct	Abundance	Water mixing, increase of turbulence	Winter NAO	[21], [23]
	SST	Direct	Abundance/distribution	Increase in water temperature	Winter SST	[66], [20]
Pelagic fish	NAO	Indirect	Abundance/food availability	Changes in temperature and wind patterns causing regime shift, changes in the pattern of transport of herring in the North Sea	Winter NAO	[67], [68]
	SST	Direct	Spawning, recruitment, distribution	Alteration of physiological rates (eggs hatching, larvae and juvenile stages)	Lag Spring SST	[51], [54], [52], [69]
Seabird	NAO	Direct	Survival, reproductive output	Increase of storm frequency influencing foraging ability or chick impacts with impacts on reproductive output	Spring NAO	[7], [70] ,[27] ,[55], [59]
	SST	Indirect	Food availability	Effect on pelagic fish	Lag Spring SST	[4]

Direct effects are manifest by correlations between climatic predictors and one of the ecological descriptors. Indirect effect links ecological descriptors to climate only through its effect on another trophic level i.e. via trophic coupling.

NAO influences water circulation and sea temperature, which can result in changes to plankton communities [21]. These changes are likely to have effects upon higher trophic levels such as fish and seabirds by influencing food availability [5] or affecting wind, rainfall and air temperature which may consequently influence seabird populations through survival, e.g. by increasing extreme events occurrence [27], [7]; or reducing prey availability within range of breeding colonies which, in turn, can affect the length of seabird foraging trips at the expense of chick survival [28].

**Figure 2:**
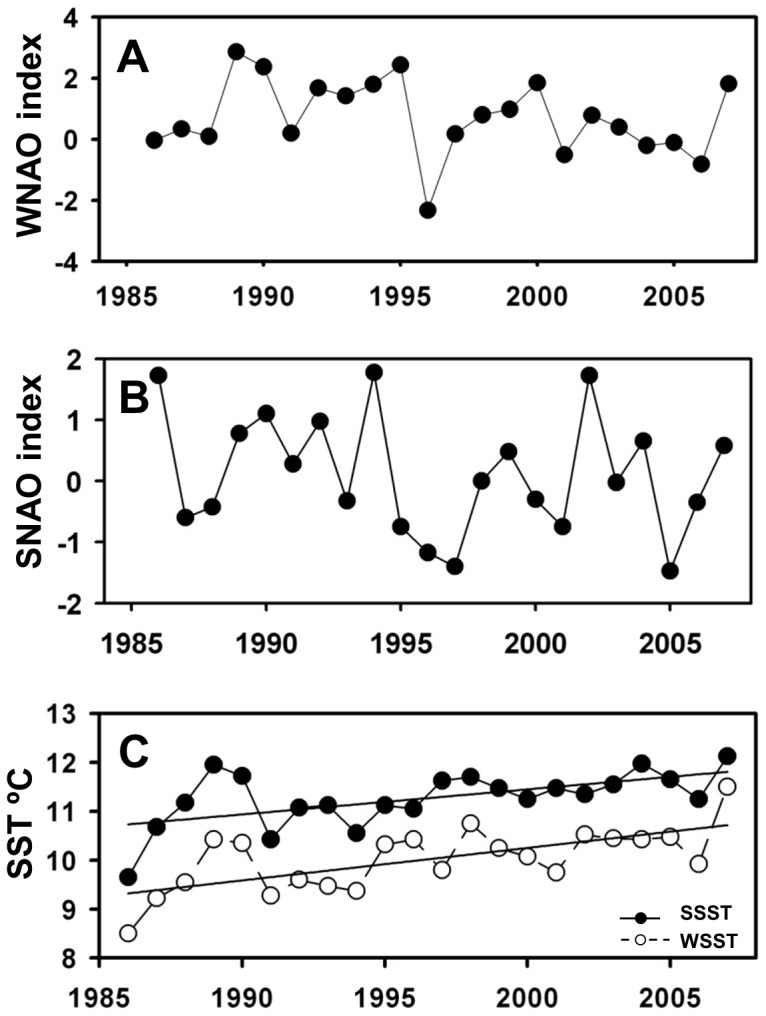
Environmental variables used for model construction. A: Winter NAO index; B: Spring NAO index; C: Sea Surface Temperature (°C). Fitted linear regressions indicate significant temporal trends.

Two different versions of the NAO index were used in this study: spring NAO during the seabird breeding season (SNAO, March–June) and winter NAO (WNAO, December–March) in order to test respectively for direct and indirect effects of climate change respectively. Monthly data from 1986 to 2007 were downloaded from the University Corporation from theAtmospheric Research (UCAR) website, Climate Analysis section http://www.cgd.ucar.edu/cas/jhurrell/indices.data.html#naostatmon) and an annual value for each index calculated (Fig. 2a, b).

Variation in SST can affect the marine ecosystem from plankton communities [23], to mid-trophic level fish [29], [9], up to apex predators such as seabirds [30] via match-mismatch events between predators and prey [12], [13]. Both winter and spring SST (WSST, December–March; SSST, March–June) were used as climate predictors. WSST was used to test for direct effects on plankton, and lagged SSST (1–2 years) to test for direct and indirect effects on pelagic fish and indirect effects on seabirds (Table 2–3). SST data were derived from satellite images and collated from the POET database available at http://poet.jpl.nasa.gov with a spatial resolution of 0.04° longitude ×0.04° latitude.

**Table 2:** Response variables and predictors used for multiple regression models.

Response Variable	Direct climate effect	Indirect climate effect	Food availability	Full model
Diatom	WNAO; WSST			WNAO+WSST+year
Small cop	WNAO; WSST		Diatom	WNAO+WSST+diatom+year
Large cop	WNAO; WSST		Diatom	WNAO+WSST+diatom+year
Her 0-g	1-lag SSST	WNAO	Small cop+large cop	WNAO+1lag-SSST+small cop+large cop+year
Her 1-g	2-lag SSST	WNAO	Small cop+large cop	WNAO+2lag-SSST+small cop+large cop+year
Kittiwake BS	SNAO	1lag-SSST	Her 0-g	SNAO+1lag-SSST+her 0-g+year
Kittiwake R_t_	SNAO	1lag-SSST	Her 0-g	SNAO+1lag-SSST+her 0-g+year
Guillemot BS	SNAO	1lag-SSST	Her 0-g+her 1-g	SNAO+1lag-SSST+her 0-g+her 1-g+year
Guillemot R_t_	SNAO	1lag-SSST	Her 0-g+her1-g	SNAO+1lag-SSST+her 0-g+her 1-g+year
Razorbill BS	SNAO	1lag-SSST	Her 0-g+her 1-g	SNAO+1lag-SSST+her 0-g+her 1-g+year
Razorbill R_t_	SNAO	1lag-SSST	Her 0-g+her1-g	SNAO+1lag-SSST+her 0-g+her 1-g+year
Puffin BS	SNAO	1lag-SSST	Her 0-g+her 1-g	SNAO+1lag-SSST+her 0-g+her 1-g+year
Puffin R_t_	SNAO	1lag-SSST	Her 0-g+her1-g	SNAO+1lag-SSST+her 0-g+her 1-g+year

For each response variable the full model is also given. WNAO: winter North Atlantic Oscillation index; SNAO: spring North Atlantic Oscillation index; WSST: winter Sea Surface Temperature; 1lag-SSST: 1 year lagged spring Sea Surface Temperature; 2lag-SSST: 2 years lagged spring Sea Surface Temperature; Small cop: small copepods (<2 mm); Large cop: large copepods (>2 mm); Her 0-g: herring 0-group; Her 1-g: herring 1-group; BS: Breeding Success expressed as the number of fledged chicks per breeding pair, weighted for sample size; R_t_: Population growth rate.

**Table 3:** Model selection to estimate factors influencing each trophic level.

Model selected	AICc weight	k	nyears	Deviance	R^2^	p-value	Slope (±Standard Error)
**PRIMARY PRODUCERS**							
**Diatom abundance**							
WSST+year	0.28	3	22	4.59	0.16	WSST 0.11**year 0.02**	WSST −0.362 (±0.221)**year 0.054 (±0.022)**
**PRIMARY CONSUMERS**							
**Small copepod biomass**							
diatom+year	0.30	3	22	1.71	0.47	diatom 0.11**year 0.003**	diatom −0.22 (±0.13)**year −0.03 (±0.01)**
**Large copepod biomass**							
diatom+WNAO	0.24	3	22	2.16	0.19	diatom 0.08WNAO 0.10	diatom −0.250 (±0.13)WNAO −0.101 (±0.06)
**SECONDARY CONSUMERS**							
**Herring 0-group abundance**							
1-lagSSST+WNAO	0.20	3	22	4.00	0.25	**1-lagSSST 0.02**WNAO 0.11	**1-lagSSST −0.450 (±0.180)**WNAO 0.133 (±0.081)
**Herring 1-group abundance**							
2-lagSSST	0.24	2	22	4.60	0.15	**0.04**	**−0.41 (±0.19)**
**APEX PREDATORS**							
**Kittiwake**							
**Productivity**							
Intercept only	0.25	1	22	1.04		<0.001	
**Population growth rate**							
SNAO	0.32	2	22	0.07	0.16	**0.036**	**0.0314 (±0.014)**
**Guillemot**							
**Productivity**							
year	0.23	2	19	0.07	0.29	**0.009**	**−0.008 (±0.002)**
**Population growth rate**							
her 1-g+year	0.45	3	22	0.07	0.12	**her 1-g 0.042**year 0.557	**her 1-g −0.352×10^−6^ (±0.161×10^−6^)**year −0.001 (±0.002)
**Razorbill**							
**Productivity**							
1lag-SSST+SNAO+her 1-g+year	0.83	5	19	0.02	0.82	**1-lagSSST 0.01** **SNAO <0.001** **her 1-g 0.003** **year 0.01**	**1-lagSSST −0.144 (±0.05)** **SNAO −0.074 (±0.013)** **her 1-g −0.884×10^−6^ (±0.167×10^−6^)** **year −0.01 (±0.003)**
**Population growth rate**							
her 1-g	0.23	2	22	0.09	0.12	her 1-g 0.09	her1-g −0.264×10^−6^ (±0.149×10^−6^)
**Puffin**							
**Productivity**							
intercept only	0.28	1	20	0.08		<0.001	
**Population growth rate**							
intercept only	0.28	1	20	0.09		0.578	

Only the best supported models are shown; AICc weight: Akaike's Information Criteria (corrected) weights, values range from 0 to 1, and high values indicate strong support for a given predictor; k: number of parameters in the model; R^2^: adjusted coefficient. WNAO: winter North Atlantic Oscillation index; SNAO: spring North Atlantic Oscillation index; WSST: winter Sea Surface Temperature; 1lag-SSST: 1 year lagged spring Sea Surface Temperature; 2lag-SSST: 2 years lagged spring Sea Surface Temperature; her 0-g: herring 0-group; her-1g: herring 1-group; Significant relationships are highlighted in **bold**; variables that are not statistically significant but feature in the best model are also presented.

### Plankton data

The Sir Alister Hardy Foundation for Ocean Science (SAHFOS) hosts the Continuous Plankton Recorder (CPR), the world's largest plankton dataset, which SAHFOS has been collecting since 1931 (for more details see [31]). This dataset represents a consistent semi-quantitative index of phytoplankton and zooplankton abundance and has captured seasonal and annual changes in plankton communities (e.g. [4], [2]).

In the present study, a total of 2299 CPR samples, taken between 1986 and 2007, were analysed to investigate possible changes in the plankton community in the Celtic Sea. The abundance of diatoms and copepods was determined in each sample and spring means calculated by averaging across samples taken in the period March–June for the Celtic Sea (49°–53°N, 4°–10°W). Copepod biomass was calculated by multiplying the abundance of each copepod taxon (mainly calanoid; 28 in total between species and taxonomic group see Table S1) by its average mass estimated from an allometric relationship based on size [31].

Diatom abundance was used as a proxy of copepod food availability [4], and copepod biomass was used as a proxy for pelagic fish prey. Copepods were divided into two groups: small copepods (<2 mm) and large copepods (>2 mm) because of changes in pelagic fish diet such as herring during different life stages. Herring larvae (8–10 mm at hatching) feed mainly on copepods and other small planktonic organisms (i.e. fish eggs) [32], [33]. Herrings are selective predators and Calanoid copepods (i.e. *Acartia* spp., *Pseudocalanus*) are the predominant prey items during the early juvenile (<3 cm) stage of life with larger herring also consuming predominantly larger copepods (i.e. *Calanus* spp.) with small fish as gape width increases [34], [35]. Smaller copepods biomass (<2 mm) was used as food proxy for young herring (1 year old, ≤10 cm Total Length (TL); hereafter 0-group), while larger size copepods biomass (>2 mm) for older herring (>1 year old, ≤15 cm TL; hereafter 1-group).

### Fish data

**Figure 3:**
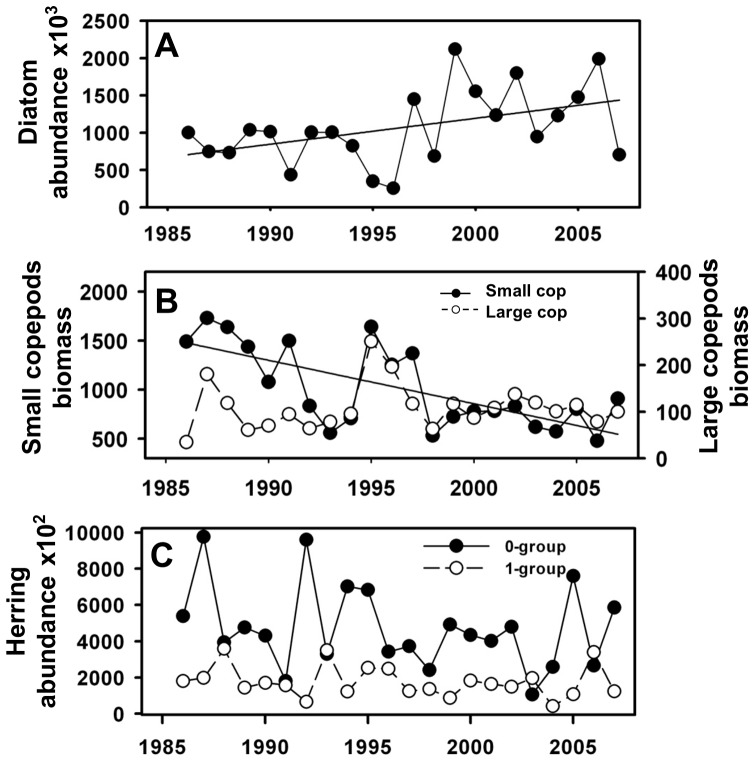
Low and mid trophic level variables used for model construction. A: diatom abundance; B: small and large copepods biomass (mg wet weight); C: Herring 0- and 1-group abundance. Fitted linear regressions indicate significant temporal trends.

Mid-trophic level fish are vectors for the transfer of energy from low trophic levels to apex predators [10]. In this study long-term data of herring 0- and 1-group abundance were used as a proxy for seabird food availability. Data spanned the period 1986–2007 and were extracted from the Herring Assessment Working Group (HAWG-ICES) acoustic survey designed to evaluate the state of pelagic fish species (sprat and herring) around the UK coast and, in particular, herring stock in the Celtic Sea ([36]; Fig. 3c).

Long-term data on other small pelagic fish such as sprat or lesser-sandeels (*Ammodytes* spp.) (hereafter, sandeel) were not available for the region. Nevertheless, data from the Centre for Environment Fisheries and Aquaculture Science (CEFAS) ground fish survey (trawl survey designed to study the distribution, composition and abundance of all fish, commercial shellfish and cephalopod species in the Celtic Sea) has shown that sprat and herring are often caught together; therefore these pelagic species can be considered ecologically equivalent (with similar habitats and diet composition) [37]. In order to test if herring was a good proxy of small pelagic fish species in the Celtic Sea, we analysed the occurrence of sprat and herring in the environment by using the only dataset available of landings from CEFAS ground fish surveys [38] covering the period 1986–2002. This showed that these two species seem to have similar fluctuations in the Celtic Sea region (Figure S1). Herring is one of the most abundant planktivorous fish in the Celtic Sea, and juvenile stages (0- and 1-group) along with other small schooling pelagic fish, such as sandeel or sprat, are an important prey of many seabird species [39]. In the Celtic Sea herring juveniles tend to remain in shallow coastal areas (nursery) for the first two years of their lives [36]. For these reasons we believe that herring (0- and 1-group) represents a good proxy for seabird food availability.

### Seabird data

**Figure 4:**
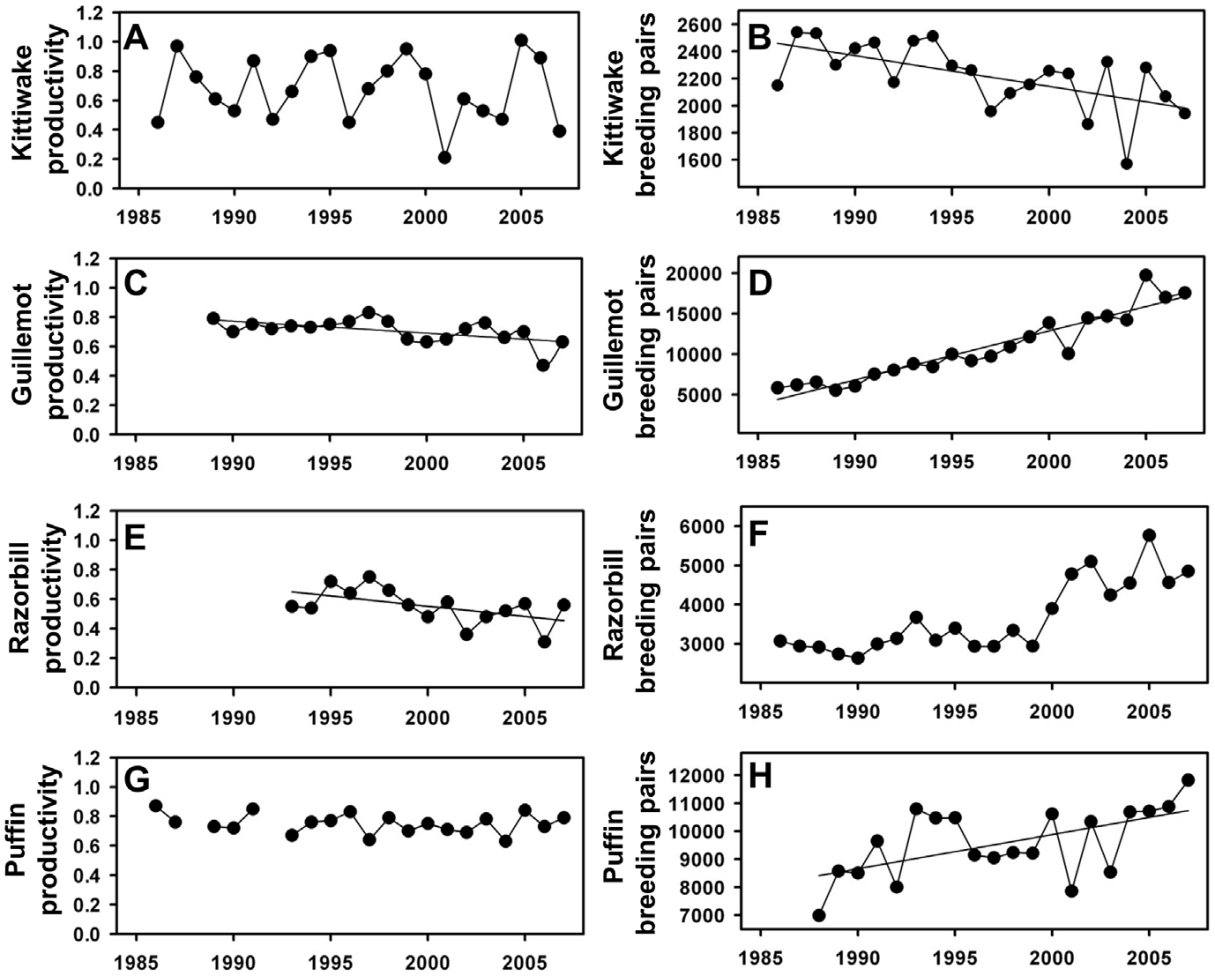
Apex predators variables used for model construction. A: kittiwake productivity (number of fledged chicks per breeding pair, weighted for sample size), and B: population count; C: guillemot productivity (number of fledged chicks per breeding pair, weighted for sample size) and D: population count; E: Razorbill productivity (number of fledged chicks per breeding pair, weighted for sample size) and F: population count; G: puffin productivity (number of fledged chicks per breeding pair, weighted for sample size) and H: population count. Fitted linear regressions indicate significant temporal trends.

Data on breeding success and population estimates for kittiwake, guillemot, razorbill and puffin breeding on Skomer Island, Wales (51°40N, 05°15W; Fig. 1) were extracted from the Seabird Monitoring Programme Database at www.defra.jncc.gov.uk/smp. These data spanned the period from 1986 to 2007 (Fig. 4) with 22 years for kittiwake, 19 for guillemot, 15 for razorbill and 20 for puffin.

These four seabirds are characterised by different foraging and reproductive strategies. Kittiwakes are surface feeders and lay an average of 3 eggs per breeding attempt, while guillemots, razorbills and puffins are all pursuit divers and lay just a single egg [15]. These four species also differ somewhat in their foraging range: kittiwake, razorbill and guillemot forage mainly inshore [40], [41], [42], whilst puffins tend to forage further offshore [43]. The species also differ in their prey loading: kittiwakes, puffins and razorbills are multiple prey-loaders whereas guillemots are single prey-loaders. Given these ecological differences and their possible diverse vulnerability to food shortage and hence climate change, we modelled their responses separately.

### Model construction and statistical analyses

Colinearity among explanatory variables may increase the probability of type I errors, therefore we tested for possible temporal trends in our data using linear regression. Multiple regression models were used to identify the main predictors for each trophic level (diatoms, copepods, herring and seabirds). When the dependent variable was not normally distributed, it was log_e_ transformed. For the four seabird species two demographic rates were used: (1) breeding success, expressed as the number of fledged chicks per breeding pair, weighted by the number of pairs sampled and (2) population growth rate (R_t_), which was calculated from the time series as log_10_(N_t+1_)-log_10_(N_t_) [44].

The following explanatory environmental variables were examined: (1) spring NAO and winter NAO to test for direct and indirect climate effects (spring NAO for seabirds, winter NAO for plankton and fish); (2) winter SST for plankton, lagged spring SST (1,2 years lag) for pelagic fish to test for direct climate effects on each age class, lagged spring SST (1 year lag) to test for indirect climate effects on seabirds. When there was a temporal trend in the dependent variable, year was included as a continuous covariate. The variables used for the model construction are shown in Table 2.

Starting from the full model, the most parsimonious model for each trophic level was selected on the basis of the lowest Akaike Information Criterion (AIC), corrected for small sample size (AICc). This approach selects the model with the best balance between bias and precision and avoids problems of, for example, multiple testing among explanatory variables ([45]. A set of candidate models was compared using difference in AIC_c_ between the top-ranked and current model (delta AIC_c_), and by calculating the AIC_c_ weight (the scaled likelihood that each model is the best description of the data; Burnham and Anderson 2002). Competing models were selected when having their AIC_c_ within 2 of the minimum [45] and are presented in Tables S3, S4, S5. Model goodness of fit was compared using the deviance and coefficient of determination (R^2^). Covariates were considered statistically significant when the p value was <0.05. Model residuals were evaluated to check for non-normality, heteroscedasticity and autocorrelation of errors. All modelling was carried out using R version 2.8 [46].

## Results

### Correlations between covariates

Preliminary explanatory analyses showed that weak correlations occurred in some cases (Table S2); however no significant correlation was found between the environmental variables (both winter and spring measures of NAO and SST) and the other covariates.

### Environmental variables

Temporal trends in environmental variables (winter NAO, spring NAO; winter and spring SST) are shown in figure 2. There was no linear trend for either spring or winter NAO over time, but there was considerable inter-annual variability. SST increased significantly over time (winter SST: p<0.001, slope = 0.006±0.01; spring SST: p = 0.004, slope = 0.05±0.01) with the minimum value in 1986 and the maximum in 2007 (8.5°C and 11.5°C for winter SST, 9.7°C and 12.1°C for spring SST respectively).

### Phytoplankton

Diatom abundance (cell count) increased over the period 1986–2007 (p = 0.03, slope = 3.47±1.53; Fig. 3a). None of the environmental covariates (winter NAO or winter SST) explained a significant amount of the variation in diatom abundance (Table 3).

### Zooplankton

Small copepod (<2 mm) biomass (mg wet weight) declined significantly over time (p = 0.003, slope = −0.03±0.01), but there was no significant trend in large copepods (>2 mm; Table 3, Fig. 3b). Neither the environmental covariates nor diatom abundance were related to zooplankton biomass (Table 3).

### Pelagic fish

Herring abundance fluctuated over time, with no clear linear trend (Fig. 3c). Both herring 0- and 1-group did not appear to be regulated by our proxies of food availability (i.e. the biomass of small and large copepods; Table 3), but there was a weak negative climate effect for both groups, with 0-group herring showing a significant negative effect of 1 year lagged spring SST (p = 0.02, slope = −0.305±0.125) and 1-group herring a significant effect of 2 year lagged spring SST (p = 0.04, slope = −0.410±0.193; Table 3).

### Seabirds

Kittiwakes showed highly variable reproductive success, ranging from 0.21 chicks pair^−1^ in 2001 to 1.01 chicks pair^−1^ in 2005 (Fig. 4a), but was not significantly correlated with any covariates (Table 3). The number of breeding pairs has declined significantly on Skomer over the last 22 years (Fig. 4b; p = 0.002, slope = −22.71±6.62, R^2^ = 0.34) and population growth rate (R_t_) was significantly positively correlated with spring NAO (p = 0.03, slope = 0.0314±0.014).

Guillemot productivity declined over time (p = 0.009, slope = −0.008±0.002, R^2^ = 0.30): the highest productivity was in 1997 (0.83 chicks pair^−1^) and the lowest in 2006 (0.47 chicks pair^−1^; Fig. 4c), but was not significantly related to any covariates (Table 3). The number of breeding pairs increased significantly on Skomer (p<0.001, slope = 425.5±95.3, R^2^ = 0.51; Fig. 4d). Guillemot population growth rate was weakly negatively correlated with 1-group herrings (p = 0.04, slope = −0.352×10^−6^±0.161×10^−6^).

Razorbill productivity declined over time (p = 0.04, slope = −0.01±0.06, R^2^ = 0.23): the highest productivity was in 1997 (0.75 chicks pair^−1^) and the lowest in 2006 (0.31chicks pair^−1^) (Fig. 4e). Razorbill productivity was significantly negatively correlated with Spring SST lagged by 1 year (p = 0.01, slope = −0.144±0.05), spring NAO (p = <0.001, slope = −0.074±0.013) and with the abundance of group 1 herring (p = 0.003, slope = −0.884×10^−6^±0.167×10^−6^). The number of breeding pairs increased on Skomer (Fig. 4f); despite this, no linear trend was found. Our most parsimonious population growth model included the intercept only (Table 3).

Puffin productivity did not show a temporal trend (p = 0.37, R^2^ = −0.009) with an annual average of 0.75 chicks pair^−1^ (Fig. 4g), although the number of breeding pairs increased significantly on Skomer over time (p = 0.008, slope = 122.2±41.8, R^2^ = 0.29; Fig. 4h). None of the explanatory covariates was related to productivity or population growth rate (Table 3).

## Discussion

Our results showed both direct and indirect effects of climate change on the Celtic Sea food web, suggesting a weak climate impact from mid-trophic levels to seabirds. In particular, we found that despite changing environmental conditions in the Celtic Sea (i.e. SST is increasing), the response of organisms differed across trophic levels. Increasing SST, for example, had negative impacts on pelagic fish (herring), but did not show any effect on copepods during the study period (1986–2007) and among apex predators, only affected razorbill productivity. Possible mechanisms and explanations for these findings, as well as comparisons with climate related patterns in other regions are discussed below.

### Direct Climate change effects on the Celtic Sea pelagic food web

Our results show that diatom abundance in the Celtic Sea also increased during the period 1986 to 2007 (Fig. 3a), although in contrast to other regions in the northeast Atlantic (i.e. Irish Sea [47] this could not be directly linked with changes in climate predictors (winter NAO and winter SST). In the North Sea a species-specific response of diatoms mean annual abundance was shown to be influenced by increasing winds and SST during the last fifty years [48], therefore further research exploring factors influencing changes in phytoplankton in the Celtic Sea could focus on additional climatic indices such as wind and precipitation.

As with the nearby Irish Sea [47], our study suggests that copepod biomass in the Celtic Sea were not significantly related to changes in SST and NAO (Table 3). As with phytoplankton abundance, this is in contrast to the North Sea where mean annual abundance of calanoid copepods abundance is positively correlated with winter NAO [20], although previous work has revealed strong regional variation in this relationship within the Northeast Atlantic [3]. Previously, Pitois and Fox (2006) [24] argued that climate change had led to a structural reorganisation of zooplankton communities in the Celtic Sea region during the period 1958–2003. Our lack of a strong climate signal in copepod biomass (Table 3) over the period 1986–2007 indicates that either these changes occurred prior to the period of our study (1986–2007), or it is only possible to detect a climate change signal over four decades [2], [49]. Previous studies have found a negative correlation between copepods biomass and rainfall [47], further highlighting the importance of investigating other measures of climatic conditions.

Previous work has shown that both NAO and SST can strongly impact upon fish growth and abundance [29], [50]. Our results did not indicate an effect of winter NAO on herring 0 and 1-group, although both age classes were negatively correlated with spring SST (Table 3). Increasing SST has been found to have both positive and negative effects on small pelagic fish in the Northeast Atlantic [51], [52], [53]. The response of herring to climate change is likely to be latitude-dependent with positive responses at high latitudes and negative at lower latitudes, such as in the Celtic Sea latitudes [54]. This negative effect is likely to be explained by the direct influence of sea temperature on herring spawning and recruitment.

We found a weak positive effect of spring NAO on black-legged kittiwake population growth rate and a weak negative effect on razorbill breeding success (Table 3). The main driver for this effect is unclear but may be related to the direct effects of wind-speed or storm-frequency [7], [55], both of which are correlated with NAO. Under this scenario strong winds associated with positive NAO phases may differentially affect species such as kittiwake and razorbill because of variations in wing shape and foraging strategies.

### Trophic coupling in the Celtic Sea

Multi-trophic level studies from the nearby North Sea [12], [4] as well as in other high latitude shelf regions [5], [30], [56], [57] have shown strong evidence for bottom-up forcing (Table 4). Given the strong increase in SST over this period in the Celtic Sea (Fig. 2c), we had anticipated bottom-up control, however, our results suggest that during 1986–2007 both the plankton community and herring in the Celtic Sea were not strongly regulated via bottom-up forcing (Table 3). Our study supports previous findings, which highlighted that regional variability in the strength of bottom-up control is common, however, and there is evidence that strong variation exists within the North Sea [39], [3].

**Table 4:** Impact of climate variability across multiple trophic levels in the North Atlantic.

Region	No. Trophic Levels examined	Groups	Climate predictor	Climate effect	Reference
North Sea	4	Seabird Pelagic fishZooplankton Phytoplankton	SST	Negative (strong bottom-up)	[12], [4]
Newfoundland	2	Seabird Pelagic fish	SST	Negative	[56]
Baltic Sea	2	Seabird Pelagic fish	NAO	Negative	[5]
Gulf of Main	2	Seabird Pelagic fish	SST	Negative	[57]
Northern Norway	2	Seabird Pelagic fish	NAO, SST	Negative	[30]

SST: Sea Surface Temperature; NAO: North Atlantic Oscillation index.

In general there was no direct evidence that herring was a limiting factor for seabirds in the Celtic Sea, instead a negative correlation between seabird demographics (guillemot R_t_ and razorbill breeding success) and herring abundance (Table 3) suggested top-down control. While we cannot exclude this possibility, an alternative explanation is that this age-class of herring may exert strong top-down effects on other pelagic fish such as sandeels that form an important part of the diet of these two Alcids. A similar trophic mechanism was previously proposed in the North Sea, where herring abundance was negatively correlated with sandeel stocks [58].

A significant negative relationship between razorbill productivity and spring SST lagged by one year (Table 3), suggests indirect bottom-up forcing, since SST is unlikely to directly impact razorbills, but may instead influence the availability of mid trophic level forage fish. Under this situation, SST might be a reliable proxy for overall abundance of forage fish, rather than herring abundance alone. It is unclear why this effect was not shown by the other seabird species, but differences in foraging range and behaviour may explain this.

## Conclusions

Previous studies have suggested a strong negative impact of climate change on seabirds elsewhere in the North Atlantic (Table 4; [4] [5]); however the situation in the Celtic Sea appears to be much less clear. Although previous works have demonstrated links between climatic conditions and seabird demographics in this region [55], [59], [60], these have mostly been connected with changes in NAO indices and are consistent with direct weather effects mediated by changes in storm frequency or wind conditions. Nevertheless, a recent study revealed a link between warmer waters in the Celtic Sea and offspring condition in Manx shearwater (*Puffinus puffinus*) [61]. Therefore, the role of climate change on the Celtic Sea remains unclear but it certainly does not appear to share the same very strong signal exhibited elsewhere in the North Atlantic. Moreover, although kittiwake numbers have decreased significantly at Skomer in the past two decades, the numbers of the three Alcids has increased (Fig. 4). However, declines in the breeding success of these Alcids are perhaps reason for concern and this could be linked with density-dependent effects.

We reiterate the call for future research to focus upon multi-trophic level, region-wide research in order to understand the ecological processes regulating marine food webs in response to climate change. However, data availability is a common limitation in this approach and there are still only a small number of studies that have used these combined long-term datasets (representing all trophic levels from plankton to apex predators) in the North Atlantic (e.g. [12], [13], [4]). However, we also urge that such ecosystem level approaches should also investigate the potential for synergistic effects of fisheries on climatic impacts. Marine ecosystems are not equally sensitive to climate change, with some regions more vulnerable than others [62]. This study has important implications for our understanding of climate change impacts on marine ecosystems and in particular on apex predators, highlighting the importance of regional variability even within a relatively small geographic area (i.e. North Sea and Celtic Sea).

## Supporting Information

Figure S1 **Herring and sprat landings (kg/km^2^) from the Western and Celtic Sea Ground Fish Survey (WCGFS) (CEFAS).** This trawl survey is designed to study the distribution, composition and abundance of all fish, commercial shellfish and cephalopod species in the Celtic Sea. Pearson's coefficient of correlation: 0.715, p value = 0.001.
(JPG)

Table S1 **Zooplankton taxa used in the study.**
(DOCX)

Table S2 **Correlation matrix (Pearson's coefficient) between covariates.** Significance is indicate as follow: pvalue<0.001 ***, pvalue,<0.01**, pvalue<0.05* SNAO: spring North Atlantic Oscillation index; WNAO: winter North Atlantic Oscillation index; SSST: spring Sea Surface Temperature; WSST: winter Sea Surface Temperature; Small cop: small copepods (<2 mm); Large cop: large copepods (>2 mm); KittBS: black-legged kittiwake productivity; GuiBS: guillemot productivity; RazBS: razorbill productivity; PufBS: puffin productivity; Her 0-g: herring 0-group; Her-1g: herring 1-group; Large cop: large copepod; Small cop: small copepods; KittRt: black-legged kittiwake population growth rate; GuiRt: guillemot population growth rate; RazRt: razorbill population growth rate; PufRt: puffin population growth rate.
(DOCX)

Table S3 **Competing models for low trophic levels.** AICc weight: Akaike's Information Criteria (corrected) weights, values range from 0 to 1, and high values indicate strong support for a given predictor; k: number of parameters in the model; R^2^: Adjusted coefficient. WNAO: winter North Atlantic Oscillation index; WSST: winter Sea Surface Temperature; Significant relationships are highlighted in **bold**, not significant variables included in the model are also presented.
(DOCX)

Table S4 **Competing models for mid trophic levels.** AICc weight: Akaike's Information Criteria (corrected) weights, values range from 0 to 1, and high values indicate strong support for a given predictor; k: number of parameters in the model; R^2^: Adjusted coefficient. WNAO: winter North Atlantic Oscillation index; 1lag-SSST: 1 year lagged spring Sea Surface Temperature; 2lag-SSST: 2 years lagged spring Sea Surface Temperature; large cop: large copepods (>2 mm); significant relationships are highlighted in **bold**, not significant variables included in the model are also presented.
(DOCX)

Table S5 **Competing models for apex predators.** AICc weight: Akaike's Information Criteria (corrected) weights, values range from 0 to 1, and high values indicate strong support for a given predictor; k: number of parameters in the model; R^2^: Adjusted coefficient. WNAO: winter North Atlantic Oscillation index; SNAO: spring North Atlantic Oscillation index; WSST: winter Sea Surface Temperature; 1lag-SSST: 1 year lagged spring Sea Surface Temperature; her 0-g: herring 0-group; her-1g: herring 1-group; Significant relationships are highlighted in **bold**, not significant variables included in the model are also presented.
(DOCX)

## References

[1] Guldberg OH, Bruno JF (2010) The Impact of Climate Change on the World's Marine Ecosystems. Science 328: 1523–1528. 20558709 10.1126/science.1189930

[2] Beaugrand G, Luczak C, Edwards M (2009) Rapid biogeographical plankton shifts in the North Atlantic Ocean. Glob Change Biol 15: 1790–1803.

[3] McGinty N, Power AM, Johnson MP (2011) Variation among northeast Atlantic regions in the responses of zooplankton to climate change: Not all areas follow the same path. J Exp Mar Bio Ecol 400: 120–131.

[4] Frederiksen M, Richardson AJ, HAllyday NC, Wanless S (2006) From plankton to top predators:bottom-up control of a marine food web across four trophic levels. J Anim Ecol 75: 1259–1268. 17032358 10.1111/j.1365-2656.2006.01148.x

[5] Osterblom H, Casini M, Olsson O, Bignert A (2006) Fish, seabirds and trophic cascades in the Baltic Sea. Mar Ecol Prog Ser 323: 233–238.

[6] Ottersen G, Planque B, Belgrano A, Post E, Reid PC, et al (2001) Ecological effects of the North Atlantic Oscillation. Oecologia 128: 1–14. 28547079 10.1007/s004420100655

[7] Frederiksen M, Daunt F, Harris MP, Wanless S (2008) The demographic impact of extreme events: stochastic weather drives survival and population dynamics in a long-lived seabird. J Anim Ecol 77: 1020–1029. 18557956 10.1111/j.1365-2656.2008.01422.x

[8] Harley CDG, Randall Hughes A, Hultgren KM, Miner BG, Sorte CJB, et al (2006) The impacts of climate change in coastal marine systems. Ecol Lett 9: 228–241. 16958887 10.1111/j.1461-0248.2005.00871.x

[9] Ottersen G, Alheit J, Drinkwater K, Friedland K, Hagen E, et al.. (2004) The response of fish populations to ocean climate fluctuations. In: press OU, editor. Marine Ecosystems and climate variation. pp. 231.

[10] Cury P, Bakun A, Crawford RJM, Jarre A, Quiñones RA, et al (2000) Small pelagics in upwelling systems: patterns of interaction and structural changes in “wasp-waist” ecosystems. ICES J Mar Sci 57: 603–618.

[11] Fauchald P, Skov H, Skern-Mauritzen M, Johns D, Tveraa T (2011) Wasp-Waist Interactions in the North Sea Ecosystem. PLoS ONE 6: e22729. 21829494 10.1371/journal.pone.0022729 PMC3145753

[12] Aebischer NJ, Coulson JC, Colebrook JM (1990) Parallel long-term trends across four marine trophic levels and weather. Nature 347: 753–755.

[13] Hunt GL, Stabeno P, Walters G, Sinclair E, Brodeur RD, et al (2002) Climate change and control of the southeastern Bering Sea pelagic ecosystem. Deep Sea Res Part II: Topical Studies in Oceanography 49: 5821–5853.

[14] Ellis JR, Lancaster JE, Cadman PS, Rogers SI (2002) The marine fauna of the Celtic Sea. In: Nunn JD, editor. Marine biodiversity in Ireland and Adjacent Waters Belfast: Ulster Museum. pp. 45–65.

[15] Mitchell PI, Newton SF, Ratcliffe N, Dunn TE, editors(2004) Seabird populations of Britain and Ireland. 511 p.

[16] Pinnegar JK, Jennings S, O'Brien CM, Polunin NVC (2002) Long- Term changes in the trophic level of the Celtic Sea fish community and fish market price distribution. J Appl Ecol 39: 377–390.

[17] Wanless S, Frederiksens M, Daunt F, Scott BE, Harris M (2007) Black-legged kittiwakes as indicators of environmental change in the North Sea: Evidence from a long term studies. Prog Oceanogr 72: 30–38.

[18] OSPAR (2000) Quality Status Report 2000 Region II - Greater North Sea. Published by OSPAR Commission 149.

[19] OSPAR (2002) Quality Status Report 2000 Region III - Celtic Seas. London 2000. 116 p.

[20] Fromentin JM, Planque B (1996) Calanus and environment in the eastern North Atlantic. Role of the North Atlantic Oscillation on *Calanus finmarchicus* and *C. helgolandicus* . Mar Ecol Prog Ser 134: 111–118.

[21] Beaugrand G, Ibanez F, Reid PC (2000) Spatial, seasonal and long-term fluctuations of plankton in relation to hydroclimatic features in the English Channel, Celtic Sea and Bay of Biscay. Mar Ecol Prog Ser 200: 93–102.

[22] ICES (2008) Celtic Sea and West of Scotland. In: ICES, editor. Advice book 5. pp. 12.

[23] Planque B, Fromentin JM (1996) Calanus and environment in the eastern North Atlantic. Spatial and temporal patterns of C. finmarchicus and C. helgolandicus. Mar Ecol Prog Ser 134: 101–109.

[24] Pitois SG, Fox CJ (2006) Long-term changes in zooplankton biomass concentration and mean size over the Northwest European shelf inferred from Continuous Plankton Recorder data. ICES J Mar Sci 63: 785–798.

[25] ICES (2007) Report of the Working Group for Regional Ecosystem Description (WGRED). Copenhagen. 153 p.

[26] Henderson PA (2007) Discrete and continuous change in the fish community of the Bristol Channel in response to climate change. J Mar Biol Assoc UK 87: 589–598.

[27] Aebisher N (1993) Immediate and delayed effects of a gale in late spring on the breeding of the shag *Phalacrocorax aristotelis* . Ibis 135: 225–232.

[28] Konarzewski M, Taylor JRE (1989) The Influence of Weather Conditions on Growth of Little Auk *Alle alle* Chicks. J Avian Biol 20: 112–116.

[29] Attrill MJ, Power M (2002) Climatic influence on a marine fish assemblage. Nature 417: 275–278. 12015600 10.1038/417275a

[30] Durant JM, Anker-Nilssen T, Stenseth NC (2003) Trophic interactions under climate fluctuations: the Atlantic puffin as an example. Proc Biol Sci 270: 1461–1466. 12965010 10.1098/rspb.2003.2397 PMC1691406

[31] Richardson AJ, Walne AW, John AWG, Jonas TD, Lindley JA, et al (2006) Using continuous plankton recorder data. Progr Oceanogr 68: 27–74.

[32] Russell FS (1976) The eggs and planktonic stages of British marine fishes; London, editor. 524 p.

[33] Daan N, Rijnsdorp AD, Overbeeke GRV (1985) Predation by North Sea herring *Clupea harengus* on eggs of plaice *Pleuronectes platessa* and cod *Gadus morhua* . Trans Am Fish Soc 114: 499–506.

[34] Blaxter JHS, Hunter JR (1982) The biology of the clupeoid fishes. 1–223 p.

[35] Arrhenius F (1996) Diet composition and food selectivity of 0-group herring (*Clupea harengus* L.) and sprat (*Sprattus sprattus* L.) in the northern Baltic Sea. ICES J Mar Sci 53: 701–712.

[36] ICES (2010) Report of the Herring Assessment Working Group for the Area South of 62n (HAWG). Copenhagen. 688 p.

[37] Voss R, Dickmann M, Schmidt JO (2009) Feeding ecology of sprat (*Sprattus sprattus*, L.) and sardine (*Sardine pilchardus* W.) larvae in the German Bight, North Sea. Oecologia 51: 117–138.

[38] Parent B (2011) Population dymanics of three small pelagic fishes in the Celtic and Irish Seas: sprat *Sprattus sprattus*, pilchard *Sardina pilachardus* and anchovy *Engraulis encrasicoulus* - Master thesis. 45 p.

[39] Frederiksen M, Edwards M, Mavor RA, Wanless S (2007) Regional and annual variation in black legged kittiwake breeding productivity is related to sea surface temperature. Mar Ecol Progr Ser 350: 137–143.

[40] Ainley DG, Ford RG, Brown ED, Suryan RM, Irons DB (2003) Prey Resources, Competition, and Geographic Structure of Kittiwake Colonies in Prince William Sound. Ecology 84: 709–723.

[41] Benvenuti S, Dall'Antonia L, Lyngs P (2001) Foraging behaviour and time allocation of chick-rearing Razorbills *Alca torda* at Græsholmen, central Baltic Sea. Ibis 143: 402–412.

[42] Clarke ED, Spear LB, McCracken ML, Marques FFC, Borchers DL, et al (2003) Validating the use of generalized additive models and at-sea surveys to estimate size and temporal trends of seabird populations. J Appl Ecol 40: 278–292.

[43] Hatch SA, Meyers PM, Mulcahy DM, Douglas DC (2000) Seasonal movements and pelagic habitat use of murres and puffins determined by satellite telemetry. The Condor 102: 145–154.

[44] Royama T (1996) Analytical Populations Dynamics. London: Chapman & Hall.

[45] Burnham KP, Anderson DR (2002) Model selection and multimodel inference: a pratical information. Theoretic Approach. New York: Springer-Verlag.

[46] Team RDC (2010) R: A language and environment for statistical computing. R Foundation for Statistical computing,Vienna, Austria.

[47] Lynam CP, Lilley MKS, Bastian T, Doyle TK, Beggs SE, et al (2011) Have jellyfish in the Irish Sea benefited from climate change and overfishing? Glob Change Biol 17: 767–782.

[48] Hinder SL, Hays GC, Edwards M, Roberts EC, Walne AW, et al (2012) Changes in marine dinoflagellate and diatom abundance under climate change. Nat Clim Chang 2: 271–275.

[49] Parmesan C, Ryrholm N, Stefanescu C, Hill JK, Thomas CD, et al (1999) Poleward shifts in geographical ranges of butterfly species associated with regional warming. Nature 399: 579–583.

[50] Rijnsdorp AD, Peck MA, Engelhard GH, Möllmann C, Pinnegar JK (2009) Resolving the effect of climate change on fish populations. ICES J Mar Sci 66: 1570–1583.

[51] Toresen R (2001) Spawning stock fluctuations and recruitment variability related to temperature for selected Herring (*Clupea harengus*) stocks in the North Atlantic. Herring. Expectations for a new millennium. Lowell Wakefield Fisheries Symposium Series. pp. 315–334.

[52] Cardinale M, Hjelm J, Casini M (2004) Effect of climate recruitment success of clupeids stocks in the North Atlantic. ICES. pp. ICES CM 2004/K:2001.

[53] Graham CT, Harrod C (2009) Implications of climate change for the fishes of the British Isles. J Fish Biol 74: 1143–1205. 20735625 10.1111/j.1095-8649.2009.02180.x

[54] Toresen R, Østvedt OJ (2000) Variation in abundance of Norwegian spring-spawning herring (*Clupea harengus*, Clupeidae) throughout the 20th century and the influence of climatic fluctuations. Fish and Fisheries 1: 231–256.

[55] Votier SC, Hatchwell BJ, Beckerman A, McCleery RH, Hunter FM, et al (2005) Oil pollution and climate have a wide scale impacts on seabirds demographics. Ecol Lett 8: 1157–1164. 21352439 10.1111/j.1461-0248.2005.00818.x

[56] Carscadden JE, Montevecchi WA, Davoren GK, Nakashima BS (2002) Trophic relationships among capelin (*Mallotus villosus*) and seabirds in a changing ecosystem. ICES J Mar Sci 59: 1027–1033.

[57] Diamond AW, Devlin CM (2003) Seabirds as Indicators of Changes in Marine Ecosystems: Ecological Monitoring on Machias Seal Island. Environmental Monitoring and Assessment 88: 153–181. 14570414 10.1023/a:1025560805788

[58] Furness R (2004) Seabird breeding failures, climate change and wind farms. Scottish Bird News 74: 18–19.

[59] Votier SC, Birkhead TR, Oro D, Trinder M, Grantham MJ, et al (2008) Recruitment and survival of immature seabirds in relation to oil spills and climate variability. J Anim Ecol 77: 974–983. 18624739 10.1111/j.1365-2656.2008.01421.x

[60] Votier SC, Hatchwell BJ, Mears M, Birkhead TR (2009) Changes in the timing of egg-laying of acolonial seabird in relation to population size and environmental conditions. Mar Ecol Progr Ser 393: 225–233.

[61] Riou S, Gray C, Brooke Md, Quillfeldt P, Masello J, et al (2011) Recent impacts of anthropogenic climate change on a higher marine predator in western Britain. Mar Ecol Progr Ser 422: 105–112.

[62] Beaugrand G, Edwards M, Brander K, Luczak C, Ibanez F (2008) Causes and projections of abrupt climate-driven ecosystem shifts in the North Atlantic. Ecol Lett 11: 1157–1168. 18647332 10.1111/j.1461-0248.2008.01218.x

[63] Edwards M, Reid P, Planque B (2001) Long-trem and regional variability of phytoplankton biomass in the Northeast Atlantic (1960–1995). ICES J Mar Sci 58: 39–49.

[64] Reid PC, Edwards M, Hunt HG, Warner AJ (1998) Phytoplankton change in the North Atlantic. Nature 391: 546–546.

[65] Richardson AJ, Shoeman DS (2004) Climate impact on plankton ecosystems in the Northeast Atlantic. Science 305: 1609. 15361622 10.1126/science.1100958

[66] Beaugrand G, Reid PC, Ibanez F, Lindley JA, Edwards M (2002) Reorganization of North Atlantic marine copepod biodiversity and climate. Science 296: 1692–1694. 12040196 10.1126/science.1071329

[67] Alheit SD, Hagen E (1997) Long-term climate forcing of European herring and sardine populations. Fish Oceanogr 6: 130–139.

[68] Corten A (1999) A proposed mechanism for the Bohuslän herring periods. ICES J Mar Sci 56: 207–220.

[69] Dickey-Collas M, Engelhard GH, Möllmann C (2007) Resolving climate impacts on fish stocks. RECLAIM report: 18.

[70] Durant JM, Hjermann DØ, Anker-Nilssen T, Beaugrand G, Mysterud A, et al (2005) Timing and abundance as key mechanisms affecting trophic interactions in variable environments. Ecol Lett 8: 952–958. 10.1111/j.1461-0248.2005.00798.x 34517678

