# Supplementary figures and images for: Influence of Climate Change and Trophic Coupling across Four Trophic Levels in the Celtic Sea
Source: PLoS One. 2012 Oct 16;7(10):e47408. doi: 10.1371/journal.pone.0047408 (PMC3472987; doi:10.1371/journal.pone.0047408)

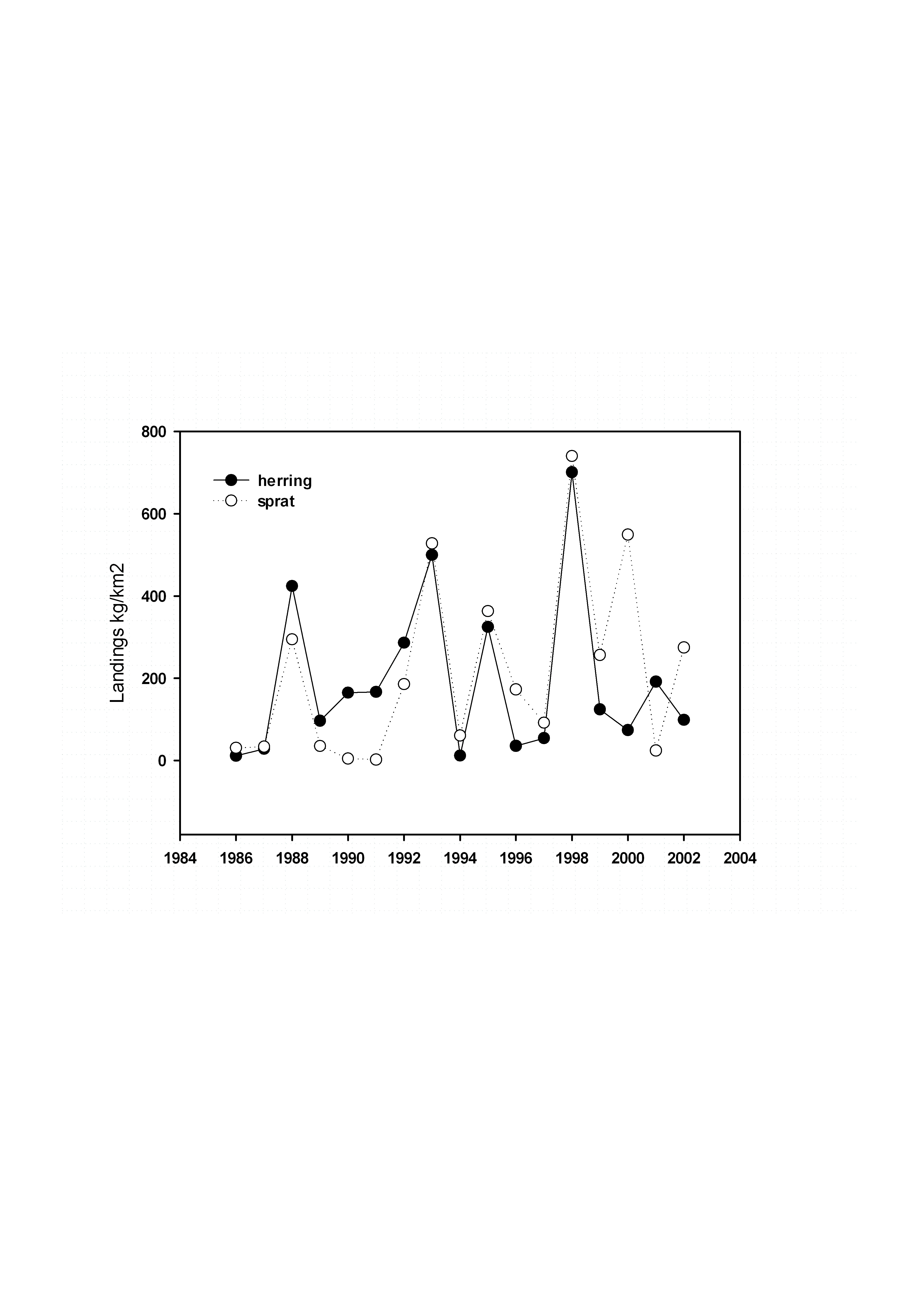

Supplement: Figure S1 — Herring and sprat landings (kg/km2) from the Western and Celtic Sea Ground Fish Survey (WCGFS) (CEFAS). This trawl survey is designed to study the distribution, composition and abundance of all fish, commercial shellfish and cephalopod species in the Celtic Sea. Pearson's coefficient of correlation: 0.715, p value = 0.001. (JPG) [file pone.0047408.s001.jpg]
