# Supplementary material for: Influence of Climate Change and Trophic Coupling across Four Trophic Levels in the Celtic Sea
Source: PLoS One. 2012 Oct 16;7(10):e47408. doi: 10.1371/journal.pone.0047408 (PMC3472987; doi:10.1371/journal.pone.0047408)
Supplement: Table S1 — Zooplankton taxa used in the study. (DOCX) [file pone.0047408.s002.docx]

|  | **Taxa** |
| --- | --- |
| 1 | traverse copepods |
| 2 | Calanus I-IV |
| 3 | Pseudocalanus elongatus Adult |
| 4 | Para-pseudocalanus spp. |
| 5 | Temora longicornis |
| 6 | Acartia spp. |
| 7 | Centropages typicus |
| 8 | Centropages hamatus |
| 9 | Isias clavipes |
| 10 | Clausocalanus spp. |
| 11 | Oithona spp. |
| 12 | Corycaeus spp. |
| 13 | Calanus Total Traverse |
| 14 | Calocalanus spp. |
| 15 | Candacia I-IV |
| 16 | Ctenocalanus vanus |
| 17 | Clytemnestra spp. |
| 18 | Harpacticoida Total |
| 19 | Euterpina acutifrons |
| 20 | Lucicutia spp. |
| 21 | Metridia I-IV |
| 22 | Metridia Total traverse |
| 23 | Oncaea spp. |
| 24 | Parapontella brevicornis |
| 25 | Scolecithricella spp. |
| 26 | Centropages chierchiae traverse |
| 27 | Metridia lucens |
| 28 | Calanus helgolandicus |

**Table S1:** Zooplankton taxa used in the study
