# Supplementary material for: Influence of Climate Change and Trophic Coupling across Four Trophic Levels in the Celtic Sea
Source: PLoS One. 2012 Oct 16;7(10):e47408. doi: 10.1371/journal.pone.0047408 (PMC3472987; doi:10.1371/journal.pone.0047408)
Supplement: Table S2 — Correlation matrix (Pearson's coefficient) between covariates. Significance is indicate as follow: pvalue<0.001 ***, pvalue,<0.01**, pvalue<0.05* SNAO: spring North Atlantic Oscillation index; WNAO: winter North Atlantic Oscillation index; SSST: spring Sea Surface Temperature; WSST: winter Sea Surface Temperature; Small cop: small copepods (<2 mm); Large cop: large copepods (>2 mm); KittBS: black-legged kittiwake productivity; GuiBS: guillemot productivity; RazBS: razorbill productivity; PufBS: puffin productivity; Her 0-g: herring 0-group; Her-1g: herring 1-group; Large cop: large copepod; Small cop: small copepods; KittRt: black-legged kittiwake population growth rate; GuiRt: guillemot population growth rate; RazRt: razorbill population growth rate; PufRt: puffin population growth rate. (DOCX) [file pone.0047408.s003.docx]

|  | **Year** | **Diatom** | **SNAO** | **WNAO** | **SSST** | **WSST** | **Kitt**  **BS** | **Guil**  **BS** | **Raz**  **BS** | **Puf**  **BS** | **Herr**  **0-gr** | **Herr**  **1-gr** | **Large**  **cop** | **Small**  **cop** | **Kitt**  **Rt** | **Gui**  **Rt** | **Raz**  **Rt** | **Puf**  **Rt** |
| --- | --- | --- | --- | --- | --- | --- | --- | --- | --- | --- | --- | --- | --- | --- | --- | --- | --- | --- |
| **Year** | 1.00 |  |  |  |  |  |  |  |  |  |  |  |  |  |  |  |  |  |
| **Diatom** | 0.36 | 1.00 |  |  |  |  |  |  |  |  |  |  |  |  |  |  |  |  |
| **SNAO** | -0.22 | 0.18 | 1.00 |  |  |  |  |  |  |  |  |  |  |  |  |  |  |  |
| **WNAO** | -0.22 | 0.09 | **0.43**  ***** | 1.00 |  |  |  |  |  |  |  |  |  |  |  |  |  |  |
| **SSST** | **0.58**  ****** | 0.26 | -0.23 | 0.21 | 1.00 |  |  |  |  |  |  |  |  |  |  |  |  |  |
| **WSST** | **0.66**  ******* | -0.001 | -0.12 | 0.17 | **0.85**  ******* | 1.00 |  |  |  |  |  |  |  |  |  |  |  |  |
| **Kitt BS** | -0.08 | 0.05 | -0.22 | 0.13 | -0.19 | -0.13 | 1.00 |  |  |  |  |  |  |  |  |  |  |  |
| **Gui BS** | **-0.59**  ****** | **-0.46**  ***** | -0.06 | 0.16 | -0.09 | -0.08 | -0.04 | 1.00 |  |  |  |  |  |  |  |  |  |  |
| **RazBS** | **-0.53**  ***** | **-0.55**  ***** | -0.49 | 0.09 | 0.08 | 0.03 | -0.01 | **0.69**  ****** | 1.00 |  |  |  |  |  |  |  |  |  |
| **Puf BS** | -0.20 | **-0.52**  ***** | -0.01 | -0.18 | **-0.50**  ***** | -0.14 | 0.13 | 0.08 | 0.11 | 1.00 |  |  |  |  |  |  |  |  |
| **Herr 0-gr** | -0.27 | -0.01 | 0.10 | 0.31 | -0.22 | -0.18 | 0.22 | 0.01 | 0.21 | 0.16 | 1.00 |  |  |  |  |  |  |  |
| **Herr 1-gr** | -0.21 | 0.23 | -0.34 | -0.19 | -0.25 | -0.24 | 0.15 | -0.20 | -0.20 | 0.08 | -0.26 | 1.00 |  |  |  |  |  |  |
| **Large cop** | -0.12 | -0.39 | -0.29 | -0.27 | -0.36 | -0.13 | 0.14 | 0.20 | 0.39 | 0.27 | 0.25 | 0.22 | 1.00 |  |  |  |  |  |
| **Small cop** | **-0.69**  ******* | **-0.49**  ***** | -0.14 | 0.04 | -0.36 | -0.32 | 0.13 | **0.52**  ***** | **0.63**  ***** | 0.33 | 0.29 | 0.20 | **0.49**  ***** | 1.00 |  |  |  |  |
| **KittRt** | 0.00 | 0.26 | **0.43**  ***** | 0.23 | 0.04 | -0.03 | -0.17 | -0.10 | -0.04 | -0.38 | 0.23 | -0.41 | -0.10 | -0.03 | 1.00 |  |  |  |
| **GuiRt** | -0.03 | 0.06 | 0.29 | -0.15 | 0.13 | -0.03 | -0.42 | 0.03 | 0.12 | -0.37 | -0.11 | -0.43 | -0.17 | -0.16 | 0.22 | 1.00 |  |  |
| **RazRt** | 0.10 | 0.27 | 0.19 | 0.02 | 0.03 | -0.15 | -0.18 | -0.29 | -0.07 | -0.35 | -0.11 | -0.41 | -0.28 | -0.20 | 0.11 | 0.37 | 1.00 |  |
| **PufRt** | -0.12 | 0.17 | -0.05 | -0.20 | 0.23 | -0.12 | -0.32 | -0.05 | 0.04 | -0.20 | 0.07 | 0.00 | -0.20 | 0.15 | 0.22 | 0.42 | 0.23 | 1.00 |

**Table S2**: Correlation matrix (Pearson’s coefficient) between covariates.

Significance is indicate as follow: pvalue< 0.001 ***, pvalue,< 0.01**, pvalue<0.05* SNAO: spring North Atlantic Oscillation index; WNAO: winter North Atlantic Oscillation index; SSST: spring Sea Surface Temperature; WSST: winter Sea Surface Temperature; Small cop: small copepods (<2mm); Large cop: large copepods (>2mm); KittBS: black-legged kittiwake productivity; GuiBS: guillemot productivity; RazBS: razorbill productivity; PufBS: puffin productivity; Her 0-g: herring 0-group; Her-1g: herring 1-group; Large cop: large copepod; Small cop: small copepods; KittRt: black-legged kittiwake population growth rate; GuiRt: guillemot population growth rate; RazRt: razorbill population growth rate; PufRt: puffin population growth rate.
