# Supplementary material for: Influence of Climate Change and Trophic Coupling across Four Trophic Levels in the Celtic Sea
Source: PLoS One. 2012 Oct 16;7(10):e47408. doi: 10.1371/journal.pone.0047408 (PMC3472987; doi:10.1371/journal.pone.0047408)
Supplement: Table S3 — Competing models for low trophic levels. AICc weight: Akaike's Information Criteria (corrected) weights, values range from 0 to 1, and high values indicate strong support for a given predictor; k: number of parameters in the model; R2: Adjusted coefficient. WNAO: winter North Atlantic Oscillation index; WSST: winter Sea Surface Temperature; Significant relationships are highlighted in bold, not significant variables included in the model are also presented. (DOCX) [file pone.0047408.s004.docx]

**Table S3** Competing models for low trophic levels.

| **Model selected** | **AICc weight** | **k** | **n**  **years** | **Deviance** | **R^2^** | **p-value** | **Slope (±Standard Error)** |
| --- | --- | --- | --- | --- | --- | --- | --- |
| **Diatom** | | | | | | | |
| WSST + year | 0.28 | 3 | 22 | 4.59 | 0.16 | WSST 0.11  **year 0.02** | WSST -0.362 (±0.221)  **year 0.054 (±0.022)** |
|  |  |  |  |  |  |  |  |
| year | 0.25 | 2 | 22 | 5.24 | 0.09 | 0.09 | 0.03 (±0.01) |
|  |  |  |  |  |  |  |  |
| **Small copepods** | | | | | | | |
| diatom + year | 0.30 | 3 | 22 | 1.71 | 0.47 | diatom 0.115  **year 0.003** | diatom -0.22 (±0.13)  **year -0.03 (±0.01)** |
| year | 0.26 | 2 | 22 | 1.96 | 0.43 | **year <0.001** | **year 0.04 (±0.01)** |
| **Large copepods** | | | | | | | |
| diatom + WNAO | 0.24 | 3 | 22 | 2.16 | 0.19 | diatom 0.08  WNAO 0.10 | diatom -0.250 (±0.13)  WNAO -0.101 (±0.06) |
| WNAO | 0.16 | 4 | 22 | 2.53 | 0.09 | WNAO 0.09 | WNAO -0.111 (±0.06) |

AICc weight: Akaike’s Information Criteria (corrected) weights, values range from 0 to 1, and high values indicate strong support for a given predictor; k: number of parameters in the model; R^2^: Adjusted coefficient. WNAO: winter North Atlantic Oscillation index; WSST: winter Sea Surface Temperature; Significant relationships are highlighted in **bold,** not significant variables included in the model are also presented.
