# Supplementary material for: Influence of Climate Change and Trophic Coupling across Four Trophic Levels in the Celtic Sea
Source: PLoS One. 2012 Oct 16;7(10):e47408. doi: 10.1371/journal.pone.0047408 (PMC3472987; doi:10.1371/journal.pone.0047408)
Supplement: Table S4 — Competing models for mid trophic levels. AICc weight: Akaike's Information Criteria (corrected) weights, values range from 0 to 1, and high values indicate strong support for a given predictor; k: number of parameters in the model; R2: Adjusted coefficient. WNAO: winter North Atlantic Oscillation index; 1lag-SSST: 1 year lagged spring Sea Surface Temperature; 2lag-SSST: 2 years lagged spring Sea Surface Temperature; large cop: large copepods (>2 mm); significant relationships are highlighted in bold, not significant variables included in the model are also presented. (DOCX) [file pone.0047408.s005.docx]

**Table S4:** Competing models for mid trophic levels.

| **Model selected** | **AICc weight** | **k** | **n**  **years** | **Deviance** | **R^2^** | **p-value** | **Slope (±Standard Error)** |
| --- | --- | --- | --- | --- | --- | --- | --- |
| **Herring 0-group** | | | | | | | |
| 1lag-SSST + WNAO | 0.20 | 3 | 22 | 4.00 | 0.25 | **1lag-SSST 0.02**  WNAO 0.11 | **1lag-SSST -0.450 (±0.180)**  WNAO 0.133(±0.081) |
| 1lag-SSST | 0.18 | 2 | 22 | 4.57 | 0.19 | **0.02** | **-0.463 (±0.188)** |
| **Herring 1-group** | | | | | | | |
| 2lag-SSST | 0.24 | 2 | 22 | 4.60 | 0.15 | **0.04** | **-0.41 (±0.19)** |
| 2lag-SSST + large cop | 0.09 | 3 | 22 | 4.44 | 0.13 | 2lag-SSST 0.08  large cop 0.43 | 2lag-SSST -0.37 (±0.20)  large cop 0.001(±0.002) |
|  |  |  |  |  |  |  |  |

AICc weight: Akaike’s Information Criteria (corrected) weights, values range from 0 to 1, and high values indicate strong support for a given predictor; k: number of parameters in the model; R^2^: Adjusted coefficient. WNAO: winter North Atlantic Oscillation index; 1lag-SSST: 1 year lagged spring Sea Surface Temperature; 2lag-SSST: 2 years lagged spring Sea Surface Temperature; large cop: large copepods (>2mm); significant relationships are highlighted in **bold**, not significant variables included in the model are also presented.
