# Supplementary material for: Influence of Climate Change and Trophic Coupling across Four Trophic Levels in the Celtic Sea
Source: PLoS One. 2012 Oct 16;7(10):e47408. doi: 10.1371/journal.pone.0047408 (PMC3472987; doi:10.1371/journal.pone.0047408)
Supplement: Table S5 — Competing models for apex predators. AICc weight: Akaike's Information Criteria (corrected) weights, values range from 0 to 1, and high values indicate strong support for a given predictor; k: number of parameters in the model; R2: Adjusted coefficient. WNAO: winter North Atlantic Oscillation index; SNAO: spring North Atlantic Oscillation index; WSST: winter Sea Surface Temperature; 1lag-SSST: 1 year lagged spring Sea Surface Temperature; her 0-g: herring 0-group; her-1g: herring 1-group; Significant relationships are highlighted in bold, not significant variables included in the model are also presented. (DOCX) [file pone.0047408.s006.docx]

**Table S5:** Competing models for apex predators.

| **Model selected** | **AICc weight** | **k** | **n**  **years** | **Deviance** | **R^2^** | **p-value** | **Slope (±Standard Error)** |
| --- | --- | --- | --- | --- | --- | --- | --- |
| **Black-legged kittiwake** | | | | | | | |
| **Productivity** | | | | | | | |
| intercept only | 0.25 | 1 | 22 | 1.04 | **--** | <0.001 |  |
| **Population growth rate** | | | | | | | |
| SNAO | 0.32 | 2 | 22 | 0.07 | 0.16 | **0.036** | **0.0314 (±0.014)** |
| SNAO + her 0-g | 0.14 | 3 | 22 | 0.07 | 0.16 | **SNAO 0.045**  her 0-g 0.349 | **SNAO 0.03 (±0.014)**  her 0-g 0.575x10^-7^ (±0.6 x10^-7^) |
| **Common guillemot** | | | | | | | |
| **Productivity** | | | | | | | |
| year | 0.23 | 2 | 19 | 0.07 | 0.29 | **0.009** | **-0.008 (±0.002)** |
| SNAO + her 1-g + year | 0.12 | 4 | 19 | 0.05 | 0.38 | SNAO 0.113  her 1-g 0.097  **year 0.003** | SNAO -0.028 (±0.017)  her 1-g -0.344x10^-6^ (±0.194x10^-6^)  **year -0.009 (±0.002)** |
| her 1-g + year | 0.12 | 3 | 19 | 0.07 | 0.31 | her 1-g 0.261  **year 0.008** | her 1-g -0.222x10^-6^ (±0.190x10^-6^)  **year -0.008 (±0.002)** |
| SNAO + year | 0.10 | 3 | 19 | 0.07 | 0.30 | SNAO 0.319  **year 0.007** | SNAO -0.017 (±0.016)  **year -0.008 (±0.002)** |
| **Population growth rate** | | | | | | | |
| her 1-g + year | 0.45 | 3 | 22 | 0.07 | 0.12 | **her 1-g 0.042**  year 0.557 | **her 1-g -0.352x10^-6^ (±0.161x10^-6^)**  year -0.001 (±0.002) |
| **Razorbill** | | | | | | | |
| **Productivity** | | | | | | | |
| 1lag-SSST + SNAO + her 1-g + year | 0.83 | 5 | 19 | 0.02 | 0.82 | **1lag-SSST 0.01**  **SNAO <0.001**  **her 1-g 0.003**  **year 0.01** | **1lag-SSST -0.144 (±0.05)**  **SNAO -0.074 (±0.013)**  **her 1-g -0.884x10-6 (±0.167x10-6)**  **year -0.01 (±0.003)** |
| **Population growth rate** | | | | | | | |
| her 1-g | 0.23 | 2 | 22 | 0.06 | 0.09 | her 1-g 0.09 | her1-g -0.264x10^-6^ (±0.149x10^-6^) |
| her 0-g + her 1-g | 0.15 | 3 | 22 | 0.06 | 0.13 | her 0-g 0.288  **her 1-g 0.005** | her 0-g -0.06x10^-6^ (±0.055x10^-6^)  her1-g -0.327x10^-6^ (±0.147x10^-6^) |
| **Atlantic puffin** | | | | | | | |
| **Productivity** | | | | | | | |
| intercept only | 0.28 | 1 | 20 | 0.08 | -- | <0.001 |  |
| **Population growth rate** | | | | | | | |
| intercept only | 0.28 | 1 | 20 | 0.09 | 0.55 | 0.578 |  |

AICc weight: Akaike’s Information Criteria (corrected) weights, values range from 0 to 1, and high values indicate strong support for a given predictor; k: number of parameters in the model; R^2^: Adjusted coefficient. WNAO: winter North Atlantic Oscillation index; SNAO: spring North Atlantic Oscillation index; WSST: winter Sea Surface Temperature; 1lag-SSST: 1 year lagged spring Sea Surface Temperature; her 0-g: herring 0-group; her-1g: herring 1-group; Significant relationships are highlighted in **bold**, not significant variables included in the model are also presented.
